# Global biogeography, cryptic species and systematic issues in the shrimp genus *Hippolyte* Leach, 1814 (Decapoda: Caridea: Hippolytidae) by multimarker analyses

**DOI:** 10.1038/s41598-017-06756-1

**Published:** 2017-07-27

**Authors:** Mariana Terossi, Sammy De Grave, Fernando L. Mantelatto

**Affiliations:** 1 Laboratory of Bioecology and Crustacean Systematics (LBSC), Department of Biology, Faculty of Philosophy, Sciences and Letters at Ribeirão Preto (FFCLRP), University of São Paulo (USP), Avenida Bandeirantes 3900, CEP 14040-901, Ribeirão Preto, São Paulo Brazil; 2 Oxford University Museum of Natural History, Parks Road, Oxford, OX1 3PW UK

## Abstract

*Hippolyte* is a genus of small bodied marine shrimps, with a global distribution. Here, we studied the phylogenetic and biogeographic relationships amongst the species of this genus with two mitochondrial and two nuclear markers, using Bayesian Inference, Maximum Likelihood, genetic divergence, molecular clock and S-DIVA. In addition, the Indo-West Pacific genus *Alcyonohippolyte* was included. Based on sequences from 57 specimens of 27 species, we recovered a robust biogeographic scenario that shows the Indo-West Pacific as the probable ancestral area of the genus *Hippolyte*, which emerged in the Paleocene, followed by dispersal in three general directions: (1) South Pacific, (2) eastern Atlantic and Mediterranean Sea and (3) Americas, the latter with a primary colonization in the eastern Pacific followed by a radiation into the western Atlantic. Our analysis reveals that the species of the *H*. *ventricosa* group do not constitute a monophyletic group and *Alcyonohippolyte* does not constitute a reciprocally monophyletic group to *Hippolyte*, with both genera herein synonimised. The relationships and systematic status of several transisthmian and Atlantic species are clarified.

## Introduction

The genus *Hippolyte* Leach, 1814 currently includes 32 species with a distribution spanning across the globe: 13 species are known from the Mediterranean Sea and the adjacent eastern Atlantic, nine species occur in the Indo-West Pacific region, four species in the western Atlantic, three species in the eastern Pacific, two species in the southern Pacific area, whilst one species is amphi-Atlantic^1–3^. The genus is considered taxonomically challenging due to the small morphological differences between species coupled with significant intra-specific variation^1^. The species from the Mediterranean Sea and adjacent eastern Atlantic were revised in 1996^1^, with further information as well as an identification key for Atlantic species in 2007^2^. However, the Indo-West Pacific species have not been thoroughly reviewed and remain challenging to identify^4^. For example, many species were, in the past, confused under the name *Hippolyte ventricosa* H. Milne Edwards, 1837, often considered as occurring across the entire Indo-West Pacific. However, the distribution of *H*. *ventricosa* was restricted to the coasts of India and Pakistan^4^ and it was postulated that records from elsewhere in the Indo-West Pacific could be undescribed species of the *H*. *ventricosa* group^2, 4^, with recently a further species in this group described^3^.

Until quite recently, the genus had an additional species, *Hippolyte commensalis* Kemp, 1925. However, this species was transferred to a new genus *Alcyonohippolyte* Marin, Chan & Okuno, 2011, together with two newly described species^5^, with three more species added to this genus later on^6, 7^. This latter genus is restricted to the Indo-West Pacific, with all species considered to be obligatory symbionts of alcyonacean soft corals (Octocorallia, Alcyonacea)^7^. Both genera are clearly very closely related, sharing some important characters, e.g. the long slender rostrum, the presence of supraorbital, hepatic and antennal teeth on the carapace and the similar shape of the mouthparts and ambulatory pereiopods^5^. Nevertheless, these genera are currently thought to be distinguished by a suite of rather minor morphological differences^5^.

Most species of the genus *Hippolyte* are small bodied, between 7 to 20 mm in length with a few European species reaching 30 to 50 mm^1, 2, 8^. Perhaps not all, but certainly in most species of this genus females reach larger sizes than males^1, 9, 10^ and are often more abundant, with the taxonomy of the genus largely based on adult female morphology^1, 2^.

No studies on the phylogenetic relationships of the closely related genera *Hippolyte* and *Alcyonohippolyte* have been published, nor has their relationship to other hippolytid genera been fully resolved. Indeed, few species of the genus *Hippolyte* have been included in the broader phylogenetic studies of Decapoda^11^ nor across caridean families^12^ or the in-depth study of the family Hippolytidae sensu lato^13^. Thus, the aim of this study was to analyze the phylogenetic and biogeographic relationships amongst the species of the genera *Hippolyte* and *Alcyonohippolyte* using two mitochondrial and two nuclear markers, based on a broad representation of species.

## Results

**Figure 1:**
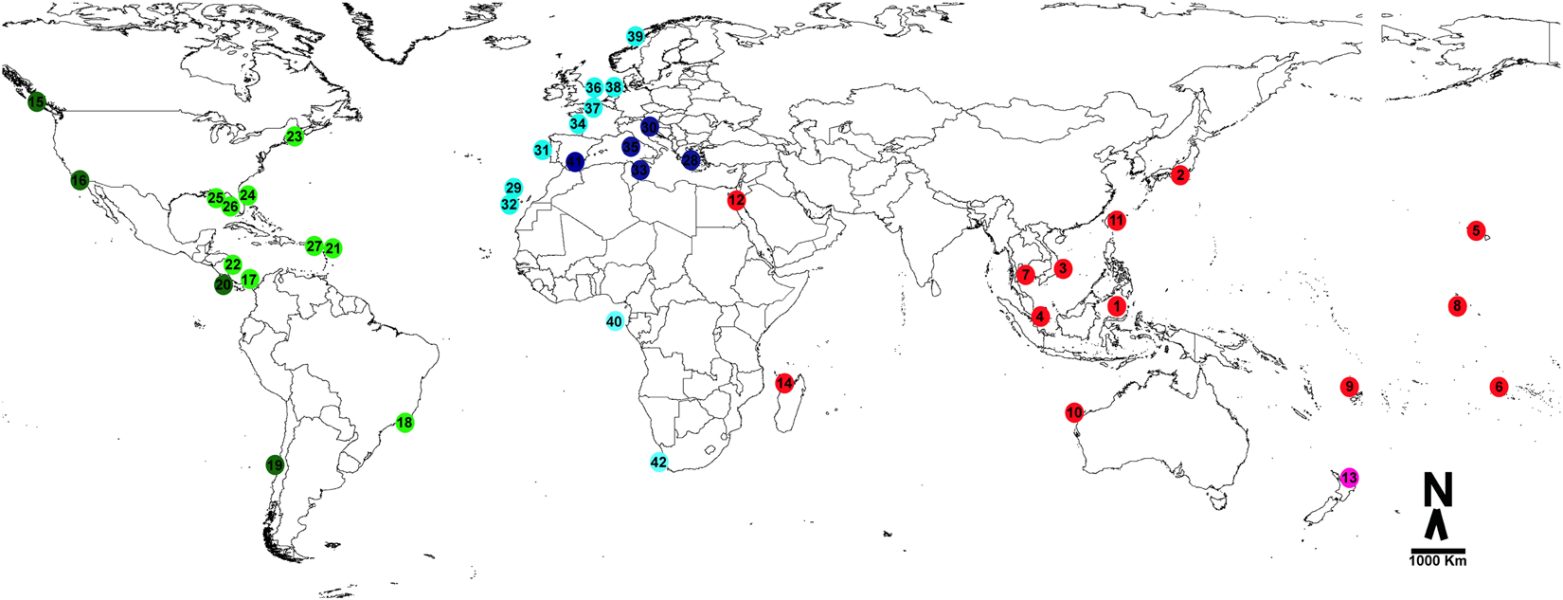
Sampling locations of specimens included in the analyses; see Supplementary Table S1 for details. The map was built with a template of the software Diva-Gis 7.5^74^ (http://www.diva-gis.org/).

For this study, specimens from 31 of the 32 species currently assigned to the genus *Hippolyte*, and four of the six species currently in the genus *Alcyonohippolyte* were available. No material was available for *Hippolyte multicolorata* Yaldwyn, 1971, *Alcyonohippolyte brachycarpus* Marin & Chan, 2012 and *Alcyonohippolyte maculata* Marin, Okuno & Chan, 2010. However, not all species were successfully amplified during the present work as some specimens were from old museum samples, possibly fixed in formaldehyde, whilst others simply did not work. This was the case for: *Hippolyte caradina* Holthuis, 1947, *Hippolyte coerulescens* (Fabricius, 1775), *Hippolyte lagarderei* d’Udekem d’Acoz, 1995, *Hippolyte leptometrae* Ledoyer, 1969, *Hippolyte palliola* Kensley, 1970, *Hippolyte proteus* (Paulson, 1875), *Hippolyte ventricosa* H. Milne Edwards, 1837 sensu stricto and *Alcyonohippolyte tenuicarpus* Marin, 2011. Thus, in the final analyses sequences from 24 *Hippolyte* species (i.e 75% of total known species diversity) and three *Alcyonohippolyte* species (i.e 50% of total known species diversity) were included with global coverage (Supplementary Table S1, Fig. 1).

### Phylogenetic analyses

All genes showed little saturation (Iss < Iss.c, p < 0.001), indicating that the data are robust enough for phylogenetic analyses. The best-fitted substitution models, selected with a corrected Bayesian information criterion were: 16S - TrN + G^14^ assumed nucleotide frequencies A = 0.3875, C = 0.0741, G = 0.1897, T = 0.3486; rate of substitution AC = 1, AG = 4.8024, AT = 1, CG = 1, CT = 14.1455, GT = 1; substitution model variable sites followed a gamma distribution with shape parameter = 0.2860; COI - TIM2 + I + G^15^ assumed nucleotide frequencies A = 0.3528, C = 0.1787, G = 0.1166, T = 0.3520; rate of substitution AC = 0.2273, AG = 4.7358, AT = 0.2273, CG = 1, CT = 7.8761, GT = 1; substitution model variable sites followed a gamma distribution with shape parameter = 0.3850; invariable sites = 0.3850; 18S - TPM2 + G^16^ assumed rate of substitution AC = 3.1084, AG = 4.8975, AT = 3.1084, CG = 1, CT = 4.8975, GT = 1; substitution model variable sites followed a gamma distribution with shape parameter = 0.1530; H3 - TrNef + I + G^15^ assumed rate of substitution AC = 1, AG = 2.6420, AT = 1, CG = 1, CT = 12.1326, GT = 1; substitution model variable sites followed a gamma distribution with shape parameter = 0.6010; invariable sites = 0.5940. We used these parameters in order to obtain the BAY tree (all markers) and the molecular clock (16S and COI).

**Figure 2:**
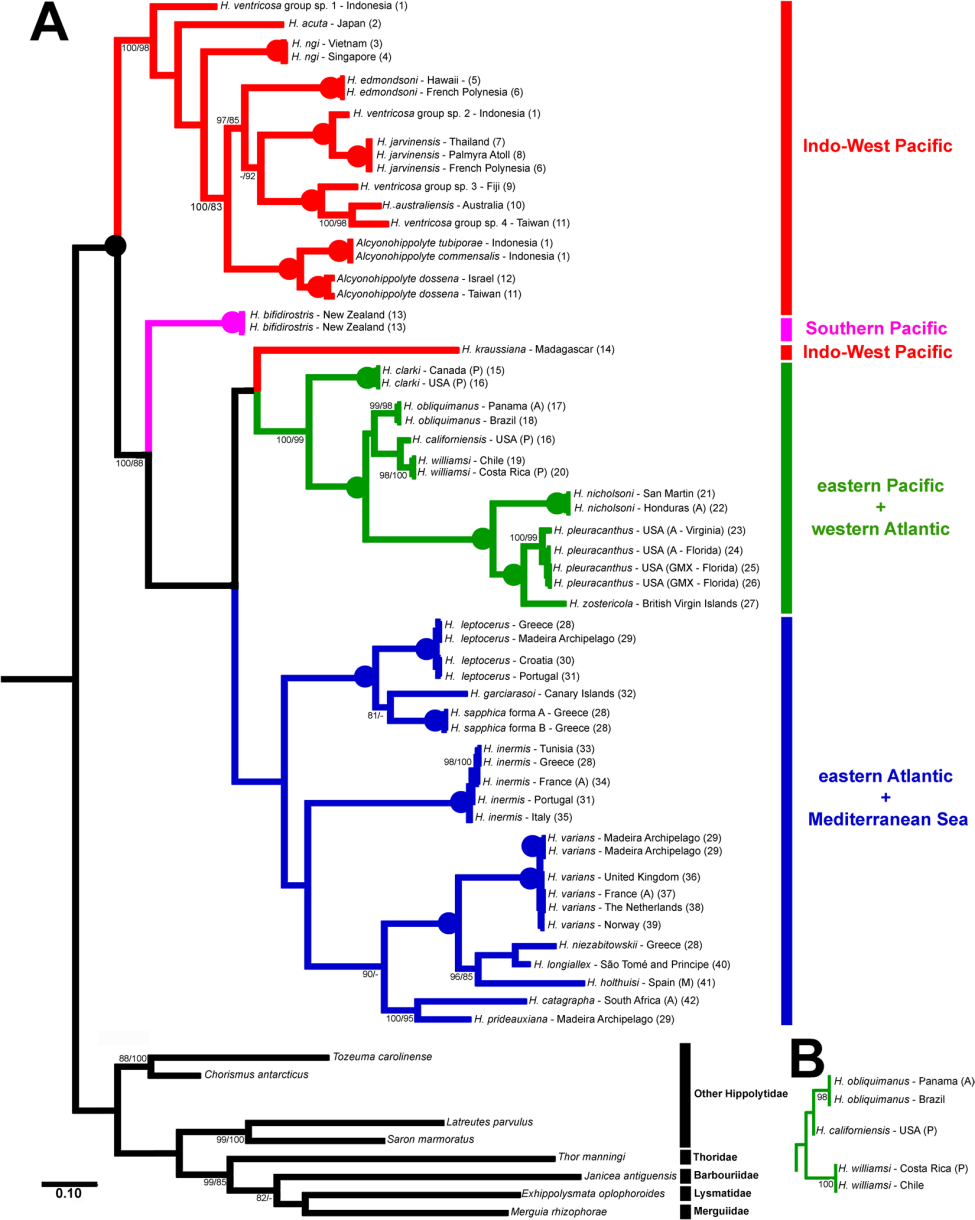
(**A**) Bayesian evolutionary tree based on combined 16S, COI, 18S and H3 DNA sequence data. Numbers at nodes represent posterior probabilities/bootstraps expressed as a percentage (BAY/ML). Numbers <80% are not shown. Fully supported (100/100) branches are marked with solid circles. The numbers between parentheses correspond to the Figure 1. Data about the specimens can be obtained in the Supplementary Table S1. (**B**) Maximum Likelihood tree showing the only topological difference found between both analyses in the position of *H*. *californiensis*, *H*. *obliquimanus* and *H*. *williamsi*. Abbreviations for seas and oceans are A: Atlantic Ocean, C: Caribbean Sea, GMX: Gulf of Mexico, M: Mediterranean Sea, P: Pacific Ocean.

We obtained almost the same topology for the phylogeny using the BAY and ML analyses. Thus, we selected the BAY tree as the basis for discussions, showing posterior probabilities (expressed as percentage), but also including bootstrap supporting values for ML analyses (Fig. 2A). The only difference between the two analyses was the relative positions of *H*. *californiensis*, *H*. *obliquimanus* and *H*. *williamsi*. In the BAY analysis, *H*. *williamsi* was the sister group of *H*. *californiensis*, and *H*. *obliquimanus* was sister group of *H*. *williamsi/H*. *californiensis* (Fig. 2A); whilst in the ML analysis, *H*. *obliquimanus* was the sister group of *H*. *californiensis*, and *H*. *williamsi* was the sister group of *H*. *obliquimanus/H*. *californiensis* (Fig. 2B). However, in both analyses, these relations (*H*. *williamsi/H*. *californiensis*, *H*. *obliquimanus/H*. *californiensis* and *H*. *williamsi/H*. *californiensis/H*. *obliquimanus*) had low support values.

The resulting trees clearly demonstrate the monophyly of the genus *Hippolyte*, and a separation into four geographically delineated clades (Fig. 2A), as follows. A basal separation splits off a clade composed of almost all Indo-West Pacific (IWP) *Hippolyte*, as well as all included *Alcyonohippolyte* species. The New Zealand species, *H*. *bifidirostris* is sister to all remaining taxa, with these further splits into two big clades, one composed almost exclusively of species from the Americas (eastern Pacific - EP and western Atlantic - WA), but with the inclusion of the Malagasy species, *H*. *kraussiana* and another comprised entirely of species from the eastern Atlantic (EA) and Mediterranean Sea (MS).

In the Indo-West Pacific clade, two results with significant systematic importance can be found (Fig. 2A): (1) the specimens of the *H*. *ventricosa* group do not constitute a monophyletic group;(2) *A*. *commensalis* and *A*. *tubiporae* constitute a single taxon.

### Genetic divergence

As expected, the mitochondrial markers were more variable than the nuclear markers (Table 1). For each of the four markers, the divergence among specimens of the genera *Alcyonohippolyte* and *Hippolyte* were of the interspecific type (Table 1). The highest divergence amongst *Alcyonohippolyte* and *Hippolyte* was lower than the highest interspecific divergence amongst *Hippolyte* species, whilst the lowest divergence amongst *Alcyonohippolyte* and *Hippolyte* was lower than the intergeneric divergence clearly placing *Alcyonohippolyte* within *Hippolyte*.

**Table 1:** Genetic divergences (%, Kimura 2-parameter) among the specimens analyzed. Data about the specimens can be obtained in the Supplementary Table S1.

Genetic divergences (%)	16S	COI	18S	H3
Intraspecific: among specimens from the same species of *Hippolyte*	0–1.1	0–4.4	0	0
Interspecific: among specimens from the different species of *Hippolyte*	2.7–29.1	14.3–41.1	0.2–14.8	0.8–13.3
Intergeneric: Among specimens from different genera (*Alcyonohippolyte* excluded)	24.3–36.6	21.5–41.1	5.2–19.2	10.0–20.3
Among specimens of *Alcyonohippolyte* and *Hippolyte*	9.4 − 27.2	18.5–32.8	3.0–14.1	4.5–11.7

For the 16S marker, the sequences of *H*. *sapphica* forms A and B showed 0% of genetic divergence, which was also the case for the specimens of *H*. *clarki* (n = 2), *H*. *edmondsoni* (n = 2), *H*. *jarvinensis* (n = 3), *H*. *nicholsoni* (n = 2), *H*. *obliquimanus* (n = 2) and *H*. *williamsi* (n = 2) analysed. The intraspecific divergence of *A*. *dossena* (n = 2), *H*. *bifidirostris* (n = 2), *H*. *leptocerus* (n = 4), *H*. *pleuracanthus* (n = 3), *H*. *varians* (n = 6) and *H*. *ngi* (n = 2) ranged from 0.3 to 0.8%, with the highest intraspecific divergence being among specimens of *H*. *inermis* (n = 5) (0–1.1%).

For the COI marker, the sequences of *H*. *sapphica* forms A and B showed 0% of genetic divergence, which was equally the case for the specimens of *H*. *clarki* (n = 2), *H*. *edmondsoni* (n = 2), *H*. *nicholsoni* (n = 2), *H*. *pleuracanthus* (n = 3) *and H*. *williamsi* (n = 2) included in the analysis. The intraspecific divergence of *H*. *obliquimanus* (n = 2) and *H*. *leptocerus* (n = 4) ranged from 0.2 to 0.4%, whilst the divergence amongst specimens of *H*. *varians* (n = 6) was 0.8%, but again the highest intraspecific divergence was amongst specimens of *H*. *inermis* (n = 5) (0.2–4.4%). The divergence between the specimen of *A*. *commensalis* and *A*. *tubiporae* respectively was 0.6%. For comparison, the divergences were 15 and 15.5% between *A*. *dossena* and *A*. *commensalis* or *A*. *tubiporae*, respectively.

For the 18S and H3 markers, the divergence was nil between the two specimens of *A*. *dossena*, and between *A*. *commensalis* and *A*. *tubiporae*, respectively. For comparison the divergences were 0.6% (18S) and 1.3% (H3) between *A*. *dossena* and both *A*. *commensalis/A*. *tubiporae*.

### Biogeographical analyses and molecular dating

**Figure 3:**
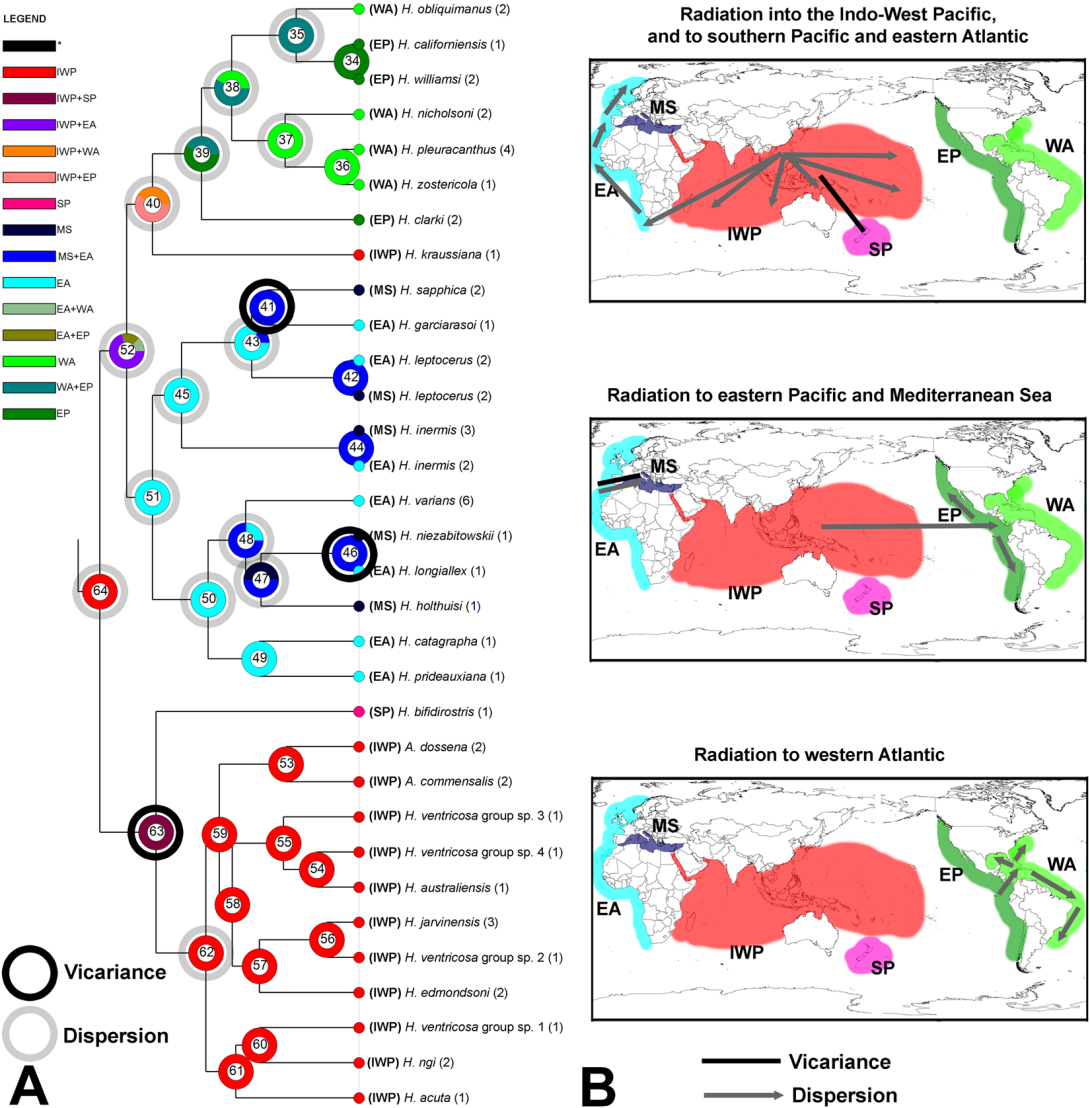
Biogeographic history of *Hippolyte*, highlighting vicariant and dispersal events. (**A**) Graphical results of ancestral distributions at each node of the phylogeny of the genus *Hippolyte* obtained by S-DIVA based on the combined 16S, COI, 18S and H3 dataset; colour key to possible ancestral ranges at different nodes; black with an asterisk represents other ancestral ranges. The numbers between parentheses correspond to the numbers of specimens. Outgroups have been excluded from the phylogeny. (**B**) Maps showing the possible geographic history of the genus. Biogeographical regions as follows: IWP: Indo-West Pacific, SP: southern Pacific, EP: eastern Pacific; WA: western Atlantic; EA: eastern Atlantic; MS: Mediterranean Sea. The maps were built with a template of the software Diva-Gis 7.5^74^ (http://www.diva-gis.org/).

**Figure 4:**
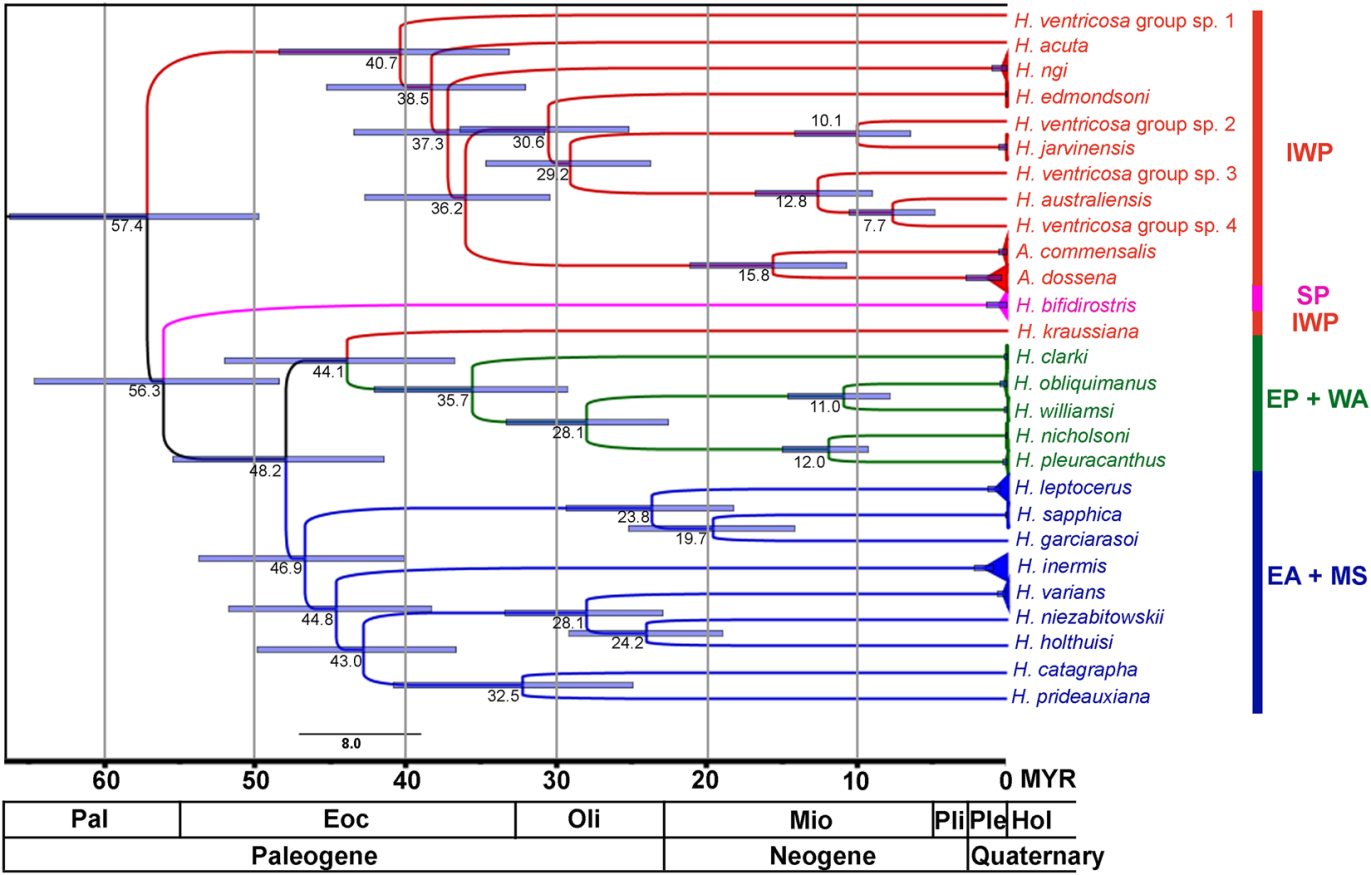
Divergence time chronogram for *Hippolyte* species estimated using a Bayesian analysis for concatenated dataset 16S/COI. Mean divergence time estimates (million years ago = MYR) are noted adjacent to their respective nodes, the bars represent the highest posterior density (95%). Geological periods of Cenozoic Era are superimposed onto the phylogeny, abbreviated as follows: Hol, Holocene; Ple, Pleistocene; Pli, Pliocene; Mio, Miocene; Oli, Oligocene; Eoc, Eocene; Pal, Paleocene. Outgroups have been excluded from the phylogeny. Biogeographical regions as in Fig. 3.

The S-DIVA results (Fig. 3) postulate that the ancestors of *Hippolyte* originated in the present day IWP (optimal area reconstruction at basal node 64) dated at 57 ± 8 Myr (Fig. 4). Node 64 indicates an early dispersion of the genus across the IWP and into the EA (probability 0.90), whilst node 63 indicates vicariance between IWP and southern Pacific (SP; probability 1.00) (Fig. 3). The separation of the clade SP dates to 56 ± 8 Myr (Fig. 4). Node 62 also indicates a further dispersion event across the IWP (probability 1.00; Fig. 3).

Possible ancestral ranges at node 52 are IWP + EA, EA + EP and EA + WA, the frequency of occurrence of these ranges being 70.23, 17.06 and 12.71%, respectively (Fig. 3), thus, the most favored ancestral range at this node being IWP + EA. This node resolves a separation, dated at 48 ± 6 Myr (Fig. 4) between a EA + MS clade with origin in EA versus a EP + WA (plus one IWP species) clade with an origin in the IWP (Fig. 3).

Node 51, dated at 47 ± 7 Myr (Fig. 4), highlights a dispersal event into the EA (probability 1.00; Fig. 3), whilst nodes 43, 45, 47, 48 and 50 (Fig. 3) suggest repeated dispersal events from EA to MS (probabilities 0.82, 0.82, 0.50, 0.38 and 0.75, respectively). Further, nodes 41 and 46 (Fig. 3) suggest vicariance events between EA and MS (probabilities 1.00 for both).

Possible ancestral ranges at node 40 are IWP + EP and IWP + WA, the frequency of occurrence of these ranges being 58.50 and 41.50% respectively (Fig. 3), thus, the most favored ancestral range at node 40 is IWP + EP (Indo-West Pacific + eastern Pacific). Also, node 40 reveals a dispersal event from IWP to EP (probability 0.90), dated 44 ± 9 Myr (Fig. 4). As node 39 (Fig. 3) is constituted by 58.5% EP and 41.5% WA + EP, possible origin is indicated for American species in the Pacific, dated at 36 ± 7 Myr (Fig. 4). Also, nodes 35, 38 and 39 reveal repeated dispersal from EP to WA (probabilities 1.00, 0.75 and 0.80, respectively) and node 37 indicates a dispersion event across the WA (probability 1.00).

## Discussion

The present analysis allows for the first time a phylogenetically based scenario for the relationships of the species of the genus *Hippolyte* and the morphologically similar *Alcyonohippolyte*, and an in depth discussion about the biogeography of this genus. Our results unambiguously demonstrate that *Alcyonohippolyte* is nested within a clade of IWP *Hippolyte* species (Fig. 2A). As our analysis included the type species of the genus, *A*. *dossena* Marin, Chan & Okuno, 2011, we herein formally consider the genus *Alcyonohippolyte* Marin, Chan & Ouno, 2011 to be a junior synonym of the genus *Hippolyte* Leach, 1814. However, it is clear that *A*. *dossena* is a distinct, valid species (Fig. 2A), within *Hippolyte* (i.e. *Hippolyte dossena* (Marin, Chan & Okuno, 2011)). A further result of the present analysis is that *Hippolyte commensalis* Kemp, 1925 (as *A*. *commensalis* in Fig. 2A) and *Alcyonohippolyte tubiporae* Marin, 2011 are clearly conspecific taxa, given that the genetic divergence between these two nominal taxa is far lower than intraspecific variation rates with the genus.

### Evolutionary biogeography

The phylogenetic tree abundantly shows the monophyly of the herein reconstituted genus *Hippolyte* and its separation into three major clades, as well as a monospecific minor clade (Fig. 2A). A complex biogeographic history is suggested by the S-DIVA analysis, which many dispersal and vicariant events shaping the current distribution pattern in the genus.

In the phylogeny, the outermost clade was formed exclusively by IWP species. The ancestral area reconstruction recovered this region as the probable ancestral area of the genus, which emerged in the Paleocene (about 57 Myr). The IWP is known as a vast tropical marine species rich hotspot^17^, with many taxa originating and diversifying in the early Cenozoic Era (65 Myr)^18–20^. This radiation has been linked to various geological events, such as the closure of the Tethys Sea, collision of Australia and proto-New Guinea with southeastern Asia, with continual changes in the availability of new tropical shallow-water habitats causing a postulated rise in the number of reefal associated taxa, such as *Hippolyte* species.

For some taxa, the IWP can be separated into three broad biogeographically distinct regions, the Indian Ocean, the Indo-Australian Archipelago and the Central West Pacific Islands^21^. For *Hippolyte*, this pattern was not observed, instead, considerable mixing of regional lineages is evident (Fig. 2A), linked to the absence of physical barriers across the region to planktonic dispersal of wide spread taxa such as *H*. *jarvinensis* and *H*. *dossena*. Clearly the presence of such taxa in peripheral areas, such as *H*. *dossena* in the Red Sea is linked to the opening up of those areas in the past^22^.

### Besides the radiation across the entire IWP region, the spread of the genus appears to have occurred in three general directions

1. South Pacific, indicated by the clade formed by *H*. *bifidirostris* [the co-distributed *H*. *multicolorata* was not included in our analysis]. Our analysis suggest an early vicariant event separating *H*. *bifidirostris* from the IWP species (Fig. 3), dated at 56 ± 8 Myr, close to the postulated origin of the genus (Fig. 4). It is worth to mention that the position of *H*. *bifidirostris* differs in the phylogenetic tree/molecular clock (outside of the EA + MS/EP + WA clades, Figs. 2A and 4) versus the S-DIVA results (outside of IWP clade, Fig. 3). As the origin of this species is very close to the herein resolved origin of the genus, it becomes difficult determine its true position, as nodes with rapid and ancient radiations are more complex to solve. In this way, although the S-DIVA suggests an early vicariant event, we are more inclined towards a dispersal scenario, as it is unclear precisely which geological event would underlie such a vicariant event.
2. Eastern Atlantic and Mediterranean Sea, indicated by a mixed geographical clade dated at 47 ± 7 Myr (Fig. 4). Our analysis suggests dispersal from the IWP into the eastern Atlantic, which such a migration around Cape of Good Hope demonstrated for fishes^22^. Our analysis also indicates repeated dispersal and vicariant events between the EA and the MS, in both directions linked to the evolution of the Tethys seaway. According to the molecular clock, the endemic Mediterranean species (*H*. *holthuisi*, *H*. *niezabitowskii* and *H*. *sapphica*) arose in the Oligocene/Miocene, thus, before the Messinian Salinity Crisis (MSC) which occurred in the Pliocene^23^. Could these species have survived the MSC? The eastern Mediterranean probably showed less drastic changes following the connection closure between the Atlantic Ocean and the Mediterranean Sea compared to the western Mediterranean^24^. A significant water body could have remained in parts of the eastern basin^24^ acting as refugia for some species. It is indeed believed that some species actually survived the MSC *in situ* ^25, 26^. Perhaps this is also the case for *Hippolyte*, especially for *H*. *sapphica*, which is endemic to the eastern Mediterranean^1^, although it cannot be ruled out that these species originated in the nearby Atlantic Ocean and migrated into the Mediterranean after the MSC.
3. Americas, indicated by the clade formed by species of the EP and WA, but with the Malagasy *H*. *kraussiana* included. The presence of *H*. *kraussiana* outside this clade is anomalous. The position of this species in the analysis is best interpreted as somewhat doubtful, as only the 16S marker could be sequenced herein, potentially influencing the analysis. According to the present analysis, the colonization of the Americas by *Hippolyte* species likely resulted from dispersal from the IWP into the EP in the Eocene, followed by a radiation into the WA. The eastern Pacific is separated from the Indo-West Pacific by a 5.000–8.000 km expanse of open ocean which has been suggested to be the world’s most effective barrier to larval dispersal^21, 27^ for the past 65 Myr^28^. However, there are examples of species and genera which have crossed this barrier, most often from west to east^21, 22, 29^, including some caridean shrimps^30^. After the colonization of the Pacific side of the Americas, it appears that the genus radiated into the western Atlantic in the Oligocene (Figs. 3 and 4).

Somewhat unexpectedly the separation of the clades of EP + WA versus EA + MS is also supported by sperm morphology. The overall sperm cell morphology of EP + WA species (*H*. *obliquimanus*, *H*. *williamsi* and *H*. *zostericola*) is very different from the EA + MS species (*H*. *inermis* and *H*. *niezabitowskii*). In the first group the sperm shows numerous posterior nuclear arms, perhaps unique within Decapoda^31, 32^, whilst in the latter clade no posterior nuclear arms are known, this being as the pattern in the majority of caridean shrimps^33, 34^.

### Cryptic or pseudocryptic species

Four specimens morphologically identified as *H*. *ventricosa* from Fiji, Indonesia, and Taiwan were included in the present analyses. These specimens all have the first article of the peduncle of the antennula with a single tooth on the outer distal corner and are different (morphologically as well as genetically) from the related taxa, *H*. *australiensis*, *H*. *edmondsoni*, *H*. *jarvinensis* and *H*. *ngi*.

Among these specimens, four genetically distinct taxa can be recognized (Fig. 2A): *H*. *ventricosa* group – sp. 1 (morphologically closest to the type specimens redescribed by d’Udekem d’Acoz in 1999^4^) and sp. 2 from Indonesia; *H*. *ventricosa* group – sp. 3 from Fiji; and *H*. *ventricosa* group – sp. 4 from Taiwan. Despite morphological similarities, these four species did not form a clade in our phylogenetic analysis (Fig. 2A), as sp. 1 was more external*;* sp. 2 is positioned close to *H*. *jarvinensis*, whilst sp. 3 and sp. 4 were positioned close to *H*. *australiensis*. Although we tissue plucked the syntypes and other material of *H*. *ventricosa* from India used in the redescription by d’Udekem d’Acoz^4^ these did not successfully amplify and no fresh material from the type locality is presently available. Hence at the moment, it is not clear which of these corresponds to the true *H*. *ventricosa*. Solving this taxonomic puzzle is beyond the present work, but it evident that several potentially new species await formal description.

### Transisthmian relationships

Three species of *Hippolyte* inhabit the eastern Pacific, *H*. *californiensis* and *H*. *clarki* are restricted to the northeastern Pacific^35^ whilst *H*. *williamsi* occurs in tropical and subtropical waters^36^. Previous studies based on morphology and molecules^37, 38^ considered the western Atlantic *H*. *obliquimanus* more closely related to *H*. *williamsi*, than to the other western Atlantic *Hippolyte*species. However up to now, *H*. *californiensis* has not been included in these comparisons. A clear morphological feature which unites these three species is the presence of up to three teeth on the outer distal corner of the first article of antennular peduncle (*H*. *californiensis* with 1–3, *H*. *obliquimanus* with 2–3, *H*. *williamsi* with 3^35, 37–39^).

Here, we analyzed specimens of *H*. *obliquimanus* from Brazil and Caribbean Panama, specimens of *H*. *williamsi* from Chile and Costa Rica and specimens of *H*. *californiensis* from the USA (Supplementary Table S1). The genetic divergence amongst these three species is very low (16S: 2.7%; COI: 14.3%; 18S: 0.2%; H3: 0.8–1.3%) compared to interspecific divergence across the genus (Table 1). In the present analyses, the only tree topology difference in the BAY and ML analyses was the positioning of these species (Fig. 2A vs. B), but support values in either analysis were low. However, the exact relationships of these taxa remain unclear as only H3 was successfully amplified for *H*. *californiensis* (Supplementary Table S1), effectively impeding fully resolved topologies. As concerns morphological data, except by two rows of subdorsal spines on pereiopods 3–5 dactylus found in *H*. *williamsi*, the variations among these species show clear overlap. Based on molecular data it is clear that all three species are valid and very closely related, but they can be distinguished by the combination of geographic distribution and some morphological characteristics. According the S-DIVA and the molecular clock analysis, there was a dispersion event between specimens from the western Atlantic (*H*. *obliquimanus*) and the eastern Pacific (*H*. *williamsi/H*. *californiensis*) in the Miocene (dated 11 ± 3 Myr), evidently before the final closure of Isthmus of Panama, dated 2.8 Myr^40^.

### Systematic issues in Atlantic taxa

*Hippolyte zostericola* was considered to be closely allied to the sympatric *H*. *pleuracanthus* by its describer^41^, with the main differentiating character being rostrum length (longer in *H*. *zostericola* than *H*. *pleuracanthus*). Although there is a considerable doubt that these species are distinct^8^, some differences among larvae of *H*. *pleuracanthus* from North Carolina (USA) and larvae of *H*. *zostericola* from Bermuda were found^42^, and there are clear colour pattern differences between both species (SDG, pers. obs.). In the present analyses, were included *H*. *zostericola* from the British Virgin Islands with long rostrum and *H*. *pleuracanthus* specimens from Florida (Atlantic Coast and Gulf of Mexico) with rostra of variable sizes. Furthermore, we expand the distribution of *H*. *pleuracanthus* now registered on both sides of the Florida peninsula (see Supplementary Table S1).

*Hippolyte sapphica* was described^43^ with two distinct morphs (A and B), separated on the basis of rostral morphology, with no known intermediate forms^1^. This species inhabits shallow seagrass meadows in the central and eastern Mediterranean^2, 44^, although forma B has only been reported from the Amvrakikos Gulf (Greece) and Venice Lagoon (Italy), always sympatric with forma A^45^. Larval development studies of both forms concluded that females of both forms can generate larvae ultimately developing in adults of either form^46^. The present phylogenetic analysis indeed corroborates that these forms belong to the same species with negligible differences in their genetic makeup.

In the present analysis (Fig. 2A), *H*. *sapphica* was closely related to *H*. *garciarasoi* and *H*. *leptocerus*, with low genetic divergence among these three species, compared to other species pairs (e.g. 18S: 0.6–0.8%; H3: 1.2–1.5%). In contrast, morphology clearly separates *H*. *sapphica* from *H*. *garciarasoi* and *H*. *leptocerus* ^2^, a difference also reflected in larval morphology^47^.

*Hippolyte garciarasoi* and *H*. *leptocerus* however share many morphological characters, with older studies having considered them to be the same species^48^, this situation was, however, resolved and both species can be clearly distinguished^49, 50^. Nevertheless, some specimens show considerable morphological variation, confounding definite assignment to either species on the basis of morphology alone^1^. However, our molecular data do indeed confirm that *H*. *garciarasoi* and *H*. *leptocerus* are two distinct species.

The specimens of *H*. *inermis* showed the highest intraspecific genetic divergence in the present dataset for the mitochondrial markers (16S and COI), with both the BAY and ML analyses showing a clear geographical division;(1) France (Atlantic Coast), Greece and Tunisia and (2) Italy and Portugal, although no morphological differences could be discerned between both groups.

*Hippolyte catagrapha* was described from False Bay (South Africa)^2^, with presumed affinities, based on morphology, to the only two other crinoid-associated species, *H*. *leptometrae* and *H*. *prideauxiana* (more northern Atlantic taxa), with within the genus only *H*. *catagrapha* and *H*. *leptometrae* having whorls of setae on the tip of their third maxilliped^2^. The present analysis (Fig. 2A) indeed considers *H*. *catagrapha* and *H*. *prideauxiana* to be sister groups. Unfortunately, the phylogenetic position of *H*. *leptometrae* remains unresolved.

*Hippolyte longiallex* was relatively recently described based on samples from São Tomé and Príncipe^2^, and considered as a probable sister species to the western Atlantic *H*. *nicholsoni*, due to shared morphological characters^2^, but perhaps more strongly suggested by the fact that both species are gorgonian commensals. The present analysis demonstrates no such phylogenetic relationship between these taxa exists, and their morphological similarity is best interpreted as an adaptation to similar hosts in these symbiotic species.

*Hippolyte niezabitowskii* from the Mediterranean Sea, is morphologically close to both *H*. *inermis* and the Mediterranean Sea *H*. *holthuisi* ^1^. Previously *H*. *holthuisi* was considered to be but a junior synonym of the Atlantic *H*. *varians*. However, morphological differences between specimens from the eastern Atlantic and the Mediterranean Sea were highlighted^1^, with specimens from Madeira being morphologically intermediate. In 1998, specimens from either side of the Strait of Gibraltar were studied^51^, an Atlantic taxon *H*. *varians* as well as the exclusively Mediterranean *H*. *holthuisi* were recognized, which was subsequently accepted in the key of the genus to species from Atlantic^2^. In the present analysis, samples of *H*. *holthuisi* were included from Mediterranean Spain, as well as a widespread sampling of Atlantic localities for *H*. *varians*. Genetics clearly demonstrates two taxa should be recognized, with *H*. *holthuisi* being phylogenetically closer to *H*. *niezabitowskii* and *H*. *longiallex* than *H*. *varians*. As for the mitochondrial markers, the divergences among specimens of *H*. *varians* were low, between specimens from Madeira versus the other localities, and it is also confirmed that specimens from this archipelago are true *H*. *varians*.

## Methods

Specimens of *Hippolyte* and *Alcyonohippolyte* were obtained from the following collections: Crustacean Collection of the Department of Biology of FFCLRP, University of São Paulo, Brazil (CCDB); Florida Museum of Natural History, USA (FLMNH); Muséum National d’Histoire Naturelle, France (MNHN); Museo de Zoología de la Universidad de Costa Rica, Costa Rica (MZ-UCR); National Museum of Natural History, Smithsonian Institution, USA (USNM); Oxford University Museum of Natural History, United Kingdom (OUMNH); Naturalis Biodiversity Centre, Leiden, the Netherlands (RMNH); University of Louisiana-Lafayette Zoological Collection, USA (ULLZ). Additional specimens were donated by Cédric d’Udekem d’Acoz (Belgium), José Cuesta and José Enrique García-Raso (Spain), Mary Wicksten (USA) and Valério Zupo (Italy); these samples were incorporated in the CCDB. All specimens were identified with the identification keys^1, 2, 8^ with reference to type and other descriptions as needed. Specimens from all regions of occurrence were examined, with global coverage (Fig. 1).

Tissue extraction, PCR amplification, product cleanup, and sequencing were conducted following previously described protocols^52^ with some modifications, mainly in product cleanup^53^. The primers and melting temperatures used were: mitochondrial 16S ribosomal gene [1472 (5′-AGATAGAAACCAACCTGG-3′)^54^, 16SL2 (5′-TGCCTGTTTATCAAAAACAT-3′)^55^, 46–48 °C]; mitochondrial Cytocrome Oxidase subunit I (COI) [COL6b (5′-ACAAATCATAAAGATATYGG-3′) and COH6 (5′-TADACTTCDGGRTGDCCAAARAAYCA-3′)^56^, COIAL2o (5′-ACGCAACGATGATTATTTTCTAC-3′) and COIAH2m (5′- GACCRAAAAATCARAATAAATGTTG-3´)^57^, 48–50 °C]; nuclear small subunit 18S ribosomal gene [18S-A (5′-AACCTGGTTGATCCTGCCAGT-3′) and 18S-L (5′-CCAACTACGAGCTTTTTAACTG-3′)^58^, 59 °C]; nuclear Histone 3 (H3) gene [H3AF (5′-ATGGCTCGTACCAAGCAGACVGC-3′) and H3AR (5′-ATATCCTTRGGCATRATRGTGAC-3′)^59^, 46–48 °C].

All sequences were confirmed by sequencing both strands (forward and reverse directions). Non-readable parts at the beginning of the sequences were omitted. All newly generated sequences were submitted to GenBank (Supplementary Table S1). Eight species were used as outgroup species, based on the phylogeny proposed in 2014^13^, some of these were retrieved from Genbank, whilst some were newly generated (Supplementary Table S1). Sequences were aligned with MUSCLE^60^, with default settings, using the online version at the Cyberinfrastructure for Phylogenetic Research (CIPRES) website^61^.

For each marker, we separately tested substitution saturation^62, 63^ in the software DAMBE 5^64^. A matrix of genetic distances was calculated under the Kimura 2-parameter (K2P) model^65^ in MEGA v5^66^ for each gene dataset (16S: 505 base pairs (bp), 59 sequences; COI: 557 bp, 46 sequences; 18S: 622 pb, 54 sequences; H3: 369 bp, 54 sequences).

A combined 16S/COI/18S/H3 dataset with 2053 bp was used for the phylogenetic analyses, with any unavailable data coded as missing and partioned by gene. The Maximum Likelihood analysis (ML) was conducted with RAxML 8.2.8^67^ using the online version at CIPRES, ML was conducted with the default parameters for RAxML for the GTR model of evolution, using the option to automatically determine the number of bootstraps to be run in RAxML^68^, thus, 900 bootstrap pseudo-replicates were run, and only confidence values >80% are reported. Prior to conducting the Inference Bayesian analysis (BAY), the model of evolution that best fitted the data was determined with the software jModelTest using BIC^69^. BAY was conducted in MrBayes v3.2.6^70^, implemented in CIPRES, with the parameters obtained from jModelTest (nucleotide frequencies, transition/transversion ratio and shape of the gamma distribution). This analysis was conducted by sampling one tree every 1,000 generations for 20,000,000 generations, starting with a random tree. Four independent BAY runs were performed and the convergence of the runs was assessed using Tracer 1.6^71^. The first 10% of parameters and trees was discarded (burn-in) and a final tree was generated in software Tree Annotator 1.8.4 (implemented in the BEAST package^72^) and visualized with FigTree 1.4.3^73^. Confidence values of posterior probabilities > 80% only are reported. The map was built with a template of the software Diva-Gis 7.5^74^.

In order to reconstruct the possible ancestral ranges of *Hippolyte* species, we used the S-DIVA^75^ analysis implemented in RASP software^76^. We used the trees generated (18,000) by our BAY run as input and defined the geographic areas as follows, based on the presence of the species in the obtained clades: IWP: Indo-West Pacific, SP: southern Pacific, EP: eastern Pacific; WA: western Atlantic; EA: eastern Atlantic; MS: Mediterranean Sea. The number of maximum ancestral areas was kept as 2. The possible ancestral ranges at each node on a selected tree were obtained. The state was sampled every 200 generations.

Divergence times within the *Hippolyte* clade were estimated using a strict clock in MrBayes implemented in CIPRES, as described above, with the same parameters obtained from jModelTest. For this analysis, only the mitochondrial markers were used and the substitution rate was fixed to 0.007 (COI) and 0.004 (16S) based on the divergence rate per one million years (Myr) reported in literature for these markers^54, 77^.

## Electronic supplementary material

Supplementary Information
