# Supplementary material for: Global biogeography, cryptic species and systematic issues in the shrimp genus Hippolyte Leach, 1814 (Decapoda: Caridea: Hippolytidae) by multimarker analyses
Source: Sci Rep. 2017 Jul 27;7:6697. doi: 10.1038/s41598-017-06756-1 (PMC5532279; doi:10.1038/s41598-017-06756-1)
Supplement: Supplementary file 1 — Supplementary Information [file 41598_2017_6756_MOESM1_ESM.pdf]

# Global biogeography, cryptic species and systematic issues in the shrimp genus *Hippolyte* Leach, 1814 (Decapoda: Caridea: Hippolytidae) by multigenes analyses

Mariana Terossi; Sammy De Grave & Fernando Luis Mantelatto

Supplementary Table S1: Specimens used in genetic analyses and respective accession numbers. Abbreviations: See Material and methods, plus UMML (University of Miami Marine Laboratories, USA). The column Map refers to the Figure 1; Accession numbers in bold were obtained from GenBank, ? – information not available.

| Species | Locality | Catalogue number | Map | 16S | COI | 18S | H3 |
|---|---|---|---|---|---|---|---|
| *Hippolyte acuta* (Stimpson, 1860) | Kanagawa, Japan | ? | 2 | **HQ315561** | - | - | - |
| *Hippolyte australiensis* (Stimpson, 1860) | Western Austrália, Australia | FLMNH 27983 | 10 | KX588880 | - | KX588921 | KX589005 |
| *Hippolyte bifidirostris* (Miers, 1876) | New Zealand | ? | 13 | **EU920927** | - | **EU920939** | **EU921063** |
| | Mission Bay, New Zealand | CCDB 6062/ OUMNH.ZC.2004-12-0001 | 13 | KX588881 | KX588969 | KX588922 | KX589006 |
| *Hippolyte californiensis* Holmes, 1895 | California,USA | CCDB 4684 | 16 | - | - | - | KX589007 |
| *Hippolyte catagrapha* d'Udekem d'Acoz, 2007 | Cape Town, South Africa | MNHN-IU-2009-3956 | 42 | KX588882 | KX588970 | KX588923 | KX589008 |
| *Hippolyte clarki* Chace, 1951 | California, USA | OUMNH.ZC.2004-14-0006 | 16 | KX588884 | KX588972 | KX588925 | KX589010 |
| | Vancouver, Canada | CCDB 6065/ OUMNH.ZC.2005-07-0006 | 15 | KX588883 | KX588971 | KX588924 | KX589009 |
| *Hippolyte edmondsoni* Hayashi, 1981 | Hekiki point, Hawai | MNHN-IU-2009-3955 | 5 | KX588885 | KX588973 | KX588926 | KX589011 |
| | Society Islands, French Polynesia | FLMNH 15559 | 6 | KX588886 | KX588974 | KX588927 | KX589012 |
| *Hippolyte garciarasoi* d'Udekem d'Acoz, 1996 | Canary Islands | RMNH 46201 | 32 | KX588890 | - | - | - |

| Species | Locality | Catalogue number | Map | 16S | COI | 18S | H3 |
|---|---|---|---|---|---|---|---|
| *Hippolyte holthuisi* Zariquiey Alvarez, 1953 | Málaga, Spain | CCDB 6048 | 41 | KX588891 | KX588978 | - | KX589018 |
| *Hippolyte inermis* Leach, 1815 | Arcachon Bay, France | OUMNH.ZC.2012-06-0009 | 34 | KX588888 | KX588976 | KX588931 | KX589016 |
| | Plataria, Greece | CCDB 2783 | 28 | JF794703 | JF794740 | KX588928 | KX589013 |
| | Lacco Ameno d´Ischia, Italy | CCDB 2383 | 35 | JF794702 | JF794739 | KX588929 | KX589014 |
| | Fuseta, Algarve, Portugal | CCDB 2779 | 31 | KX588887 | KX588975 | KX588930 | KX589015 |
| | off Sidi bou said, Tunisia | USNM 1182901 | 33 | KX588889 | KX588977 | KX588932 | KX589017 |
| *Hippolyte jarvinensis* Hayashi, 1981 | Line Islands, Palmyra Atoll | FLMNH 13794 | 8 | KX588892 | - | KX588933 | KX589019 |
| | Society Islands, French Polynesia | FLMNH 16275 | 6 | KX588893 | - | KX588934 | KX589020 |
| | Surat Thani, Thailand | RMNH 42439 | 7 | KX588894 | - | KX588935 | KX589021 |
| *Hippolyte kraussiana* (Stimpson, 1860) | Nosy Be, Madagascar | FLMNH 14144 | 14 | KX588895 | - | - | - |
| *Hippolyte leptocerus* (Heller, 1862) | Algarve, Portugal | CCDB 2777 | 31 | KX588898 | KX588981 | KX588938 | KX589024 |
| | Amvrakikos Gulf, Greece | CCDB 2780 | 28 | KX588897 | KX588980 | KX588937 | KX589023 |
| | Santa Cruz, Madeira Archipelago | RMNH 46967 | 29 | KX588899 | KX588982 | KX588939 | KX589025 |
| | Rovinj, Croatia | RMNH 46964 | 30 | KX588896 | KX588979 | KX588936 | KX589022 |
| *Hippolyte longiallex* d'Udekem d'Acoz, 2007 | São Tomé and Príncipe | MNHN-IU-2009-3961 | 40 | - | - | KX588940 | KX589026 |

| Species | Locality | Catalogue number | Map | 16S | COI | 18S | H3 |
|---|---|---|---|---|---|---|---|
| *Hippolyte ngi* Gan & Li, 2017 | Singapore | OUMNH.ZC.2014-11-286 | 4 | KX588912 | - | KX588957 | KX589043 |
| | Nhatrang Bay, Vietnam | CCDB 6063/ OUMNH.ZC.2007-18-0004 | 3 | KX588913 | - | KX588958 | KX589044 |
| *Hippolyte nicholsoni* Chace, 1972 | Cayos Grande, Honduras | CCDB 6059/OUMNH.ZC.2004-17-0002 | 22 | KX588901 | KX588984 | KX588942 | KX589028 |
| | French Antilles, St Martin | FLMNH 32258 | 21 | KX588900 | KX588983 | KX588941 | KX589027 |
| *Hippolyte niezabitowskii* d'Udekem d'Acoz, 1996 | Ionian Sea, Greece | CCDB 2776 | 28 | KX588902 | KX588985 | KX588943 | KX589029 |
| *Hippolyte obliquimanus* Dana, 1852 | São Paulo, Brazil | CCDB 2147 | 18 | JF794696 | JF794728 | KX588945 | KX589031 |
| | Bocas del Toro, Panama | CCDB 2580 | 17 | JF794692 | JF794710 | KX588944 | KX589030 |
| *Hippolyte pleuracanthus* (Stimpson, 1871) | Chesapeake Bay, Virginia, USA | ? | 23 | - | - | **AY743956** | - |
| | St. Joseph Peninsula State Park, Florida, USA | CCDB 174 | 25 | KX588903 | KX588986 | KX588946 | KX589032 |
| | Cedar Keys, Florida, USA | FLMNH 14772 | 26 | KX588904 | KX588987 | KX588947 | KX589033 |
| | Fort Pierce, Florida, USA | CCDB 6067/ OUMNH.ZC.2009-05-0010 | 24 | KX588905 | KX588988 | KX588948 | KX589034 |
| *Hippolyte prideauxiana* Leach, 1817 | Madeira Archipelago | CCDB 6068 | 29 | KX588906 | KX588989 | KX588949 | KX589035 |

| Species | Locality | Catalogue number | Map | 16S | COI | 18S | H3 |
|---|---|---|---|---|---|---|---|
| *Hippolyte sapphica* d'Udekem d'Acoz, 1993 form A | Amvrakikos Gulf, Greece | CCDB 2782 | 28 | KX588907 | KX588990 | KX588950 | KX589036 |
| *Hippolyte sapphica* d'Udekem d'Acoz, 1993 form B | Amvrakikos Guf, Greece | RMNH 46970 | 28 | KX588908 | KX588991 | KX588951 | KX589037 |
| *Hippolyte varians* Leach, 1814 | Tromsø, Sommarøy, Norway | MNHN-IU-2009-3972 | 39 | KX588909 | KX588993 | KX588952 | KX589038 |
| | Harwich, United Kingdom | ULLZ 6970 | 36 | **EU868662** | - | **EU868753** | - |
| | The Netherlands | CCDB 2781 | 38 | JF794700 | KX588993 | KX588953 | KX589039 |
| | Normandy, France | CCDB 2778 | 37 | JF794701 | KX588994 | KX588954 | KX589040 |
| | Caniço, Madeira Archipelago | RMNH 46905 | 29 | KX588910-1 | KX588995-6 | KX588955-6 | KX589041-2 |
| *H. ventricosa* group – sp. 1 | Sulawesi Sea, Indonesia | CCDB 6064/ OUMNH.ZC.2009-04-0001 | 1 | KX588914 | - | - | KX589045 |
| *H. ventricosa* group – sp. 2 | Sulawesi Sea, Indonesia | CCDB 6060/ OUMNH.ZC.2007-22-0009 | 1 | KX588915 | KX588997 | KX588959 | KX589046 |
| *H. ventricosa* group – sp. 3 | Fiji | OUMNH.ZC.2005-09-047 | 9 | KX588917 | - | - | - |
| *H. ventricosa* group – sp. 4 | Taiwan | OUMNH.ZC.2010-02-0046 | 11 | KX588916 | KX588998 | - | - |

| Species | Locality | Catalogue number | Map | 16S | COI | 18S | H3 |
|---|---|---|---|---|---|---|---|
| *Hippolyte williamsi* Schmitt, 1924 | Guanacaste, Costa Rica | MZ-UCR 2295-02 | 20 | KX588918 | KX588999 | KX588961 | KX589048 |
| | Coquimbo, Chile | CCDB 2382 | 19 | JF794699 | JF794738 | KX588960 | KX589047 |
| *Hippolyte zostericola* (Smith, 1873) | British Virgin Islands | CCDB 6061/ OUMNH.ZC.2003-33-0047 | 27 | - | - | KX588962 | - |
| *Alcyonohippolyte commensalis* (Kemp, 1925) | Indonesia | RMNH 57005 | 1 | - | KX589001 | KX588964 | KX589050 |
| *Alcyonohippolyte dossena* Marin, Okuno & Chan, 2010 | Eilat, Israel | OUMNH.ZC.2011-05-0060 | 12 | KX588919 | KX589002 | KX588965 | KX589051 |
| | Pingtung, Taiwan | OUMNH.ZC.2010-02-0067 | 11 | KX588920 | - | KX588966 | KX589052 |
| *Alcyonohippolyte tubiporae* Marin, 2011 | Indonesia | RMNH 57004 | 1 | - | KX589000 | KX588963 | KX589049 |
| *Chorismus antarticus* (Pfeffer, 1887) | Antarctica | ? | - | **FJ434340** | **EF407581** | - | - |
| *Exhippolysmata oplophoroides* (Holthuis, 1948) | São Paulo, Brazil | CCDB 3769 | - | KU312980 | KU313009 | KX588967 | - |
| *Janicea antiguensis* (Chace, 1972) | Cape Verde | OUMNH.ZC.2004-15-002 | - | **KF023112** | - | **JF346262** | **JF346333** |
| *Latreutes parvulus* (Stimpson, 1871) | São Paulo, Brazil | CCDB 3807 | - | KU312983 | KX589003 | KX588968 | KX589053 |
| *Merguia rhizophorae* (Rathbun, 1900) | Panama | OUMNH.ZC.2009-06-05 UMML 329471 | - | **EU861508** | **KC962214** | - | **KF178857** |
| *Saron marmoratus* (Olivier, 1811) | Taiwan | MNHN-IU-2012-1041 | - | **KP725649** | **KP759508** | **KP725848** | **KP726204** |
| *Thor manningi* Chace, 1972 | São Paulo, Brazil | CCDB 2737 | - | KU312995 | KX589004 | - | KX589054 |
| *Tozeuma carolinense* Kingsley, 1878 | Bocas del Toro, Panama | CCDB 2738 | - | KU312994 | - | | |
